# Evaluation on antioxidant properties of sixteen plant species from Jeju Island in Korea

**DOI:** 10.17179/excli2014-589

**Published:** 2015-01-26

**Authors:** Eun-Yi Ko, Daekyung Kim, Seong Woon Roh, Weon-Jong Yoonc, You-Jin Jeon, Ginnae Ahn, Kil-Nam Kim

**Affiliations:** 1 Jeju Center, Korea Basic Science Institute (KBSI), Jeju 690-140, Republic of Korea; 2 Department of Marine Life Science, Jeju National University, Jeju 690-756, Republic of Korea; 3 Jeju Biodiversity Research Institute, Jeju Technopark, Jeju, 699-943, Republic of Korea; 4 Department of Marine Bio-Food Sciences, Chonnam National University, Yeosu 550-749, Republic of Korea

**Keywords:** Antioxidant, plant extracts, ethyl acetate fraction, oxidative damage, phenolic compounds

## Abstract

In this study, the antioxidant properties of 80 % ethanol extracts of 16 species of plants from Jeju Island in Korea were evaluated using various antioxidant assays, including the DPPH (1,1-Diphenyl-2-pricrylhydrazyl) radical scavenging, superoxide scavenging, xanthine oxidase inhibition and hydrogen peroxide scavenging activities. Among the 16 plant extracts tested, CN-13 showed strong antioxidant properties in the DPPH radical scavenging and hydrogen peroxide scavenging tests. The CN-13 ethanol extract was thus selected to be used for further experiments, and was separated into various fractions using four different organic solvents (*n*-hexane, methylene chloride, ethyl acetate and butanol). The ethyl acetate fraction of CN-13 extract evidenced strong DPPH radical scavenging properties as compared to the other fractions. The ethyl acetate fraction also strongly inhibited DNA-damage induced by hydrogen peroxide-oxidative damage in a mouse lymphoma (L5178Y-R) cell line. Moreover, a correlation between the total phenolic content of the extract, and its antioxidant property was reported.

## Introduction

Reactive oxygen species (ROS), particularly the superoxide anion radical (^•^O_2_^-^), hydroxyl radical (^•^OH), and hydrogen peroxide (H_2_O_2_), are unwanted metabolic by-products of normal aerobic metabolism. Generally, the production of appropriate ROS that is controlled by the antioxidant system in living organisms are essential for many cellular functions such as killing phagocytes, bacterial ingestion and redox regulation of signal transduction. However, high ROS levels have been implicated in a variety of pathological conditions, including cardiovascular disease, cancer and aging (Harman, 1994[16]; Cox and Cohen, 1996[10]; Ames, 1998[3]; Finkel and Holbrook, 2000[11]). H_2_O_2_ is one of the most crucial varieties of ROS, as it is generated from nearly all sources of oxidative stress, and has the ability to diffuse freely in and out of cells and tissues (Halliwell and Aruoma, 1991[14]).

Some synthetic antioxidants have been reported to function as mutagens and tumor promoters at high dosages (Kahl and Kappus, 1993[26]; Kahl, 1994[25]). Natural antioxidants, on the other hand, impart a greater degree of safety, even at higher dosages, and such compounds may also impart other health benefits. Natural antioxidants can protect the human body against free radicals, and have also been shown to retard the progress of a variety of chronic diseases (cancer, heart disease and diabetes etc.), as well as ameliorating or retarding lipid rancidity in foods (Kinsella et al., 1993[29]). Among the various natural antioxidants, phenolic compounds are reported to have the ability to quench oxygen-derived free radicals by donating a hydrogen atom or an electron to the free radical (Wanasundara and Shahidi, 1996[49]; Yuting et al., 1990[52]). Furthermore, phenolic compounds from plant materials have been shown to neutralize free radicals in various model systems (Zhang et al., 1996[53]). Thus, recent studies have demonstrated the potential of plant products to be used as antioxidants against various diseases induced by free radicals (Hou et al., 2003[21]). Additionally, it has been determined that the antioxidant properties of plant products are mainly attributed to the presence of phenolic compounds, such as flavonoids, phenolic acids, tannins, and phenolic diterpenes (Pietta, 2000[36]).

The objective of the present study was to compare the free radical scavenging properties as well as the total phenolic contents of 80 % ethanolic extracts of 16 plant species from Jeju Island in Korea.

## Materials and Methods

### Chemicals

1,1-Diphenyl-2-pricrylhydrazyl (DPPH), peroxidase, 2,20-azino-bis (3-ethylbenzthia-zoline)-6-sulfonic acid (ABTS), xanthine oxidase, nitroblue terazolium (NBT), tannin acid, rutine, allopurinol, butylated hydroxyl anisole (BHT), trolox, 5,5-Dimethyl-1-pyrrolin *N*-oxide (DMPO), α-(4-pyridyl-1-oxide)-*N*-t-butylnitrone (4-POBN) and 2,2-azobs (2-amidinopropane) hydrochloride (AAPH) were purchased from Sigma Chemical Co. (St. Louis, MO, USA). All other chemicals used were of 99 % or greater purity.

### Preparation of plant extract and fractions

Sixteen plant species (Table 1(Tab. 1)) were collected along the Jeju Island of Korea during a period extending from February to August 2006. The collected plants were cleaned, dried under shade at room temperature and powdered. A powdered sample (300 g) of each plant was treated with 1000 ml ethanol (80 %) at room temperature with shaking. This procedure was repeated at least three times until the extraction solvent became colorless. Then extracts were filtered using Whatman No. 2 filter paper and the filtrate was collected. A rotary evaporator was used to remove the ethanol at 50 °C. The ethanolic extract was dissolved in water and separated with equal volumes of n-hexane. The residue was fractionated by a series of solvents, namely methylene chloride (CH_2_Cl_2_), ethyl acetate (EtOAc) and butanol (BuOH). Five fractions were prepared from 80 % ethanol extracts of CN-13.

### DPPH radical scavenging assay

Free radical scavenging activity was determined by using a stable free radical, DPPH, and the assay was conducted according to a slightly modified method described by Blois (1958[6]). A DPPH solution was prepared at a concentration of 4 × 10^-4^ M in methanol. During the assay, a 100-µl sample extract and a 100-µl solution of freshly prepared DPPH were thoroughly mixed. The reaction mixture was incubated at room temperature for 20 min. The absorbance was then recorded at 517 nm using an ELISA reader. The concentration of extract that provided 50 % inhibition (IC_50_) of the DPPH free radical was calculated from a plot of the inhibition percentage against the extract concentration. The experiments were carried out in triplicate. BHT, Trolox and allopurinol were used as positive controls. 

### Superoxide radical scavenging assay

Superoxide radicals were generated by the xanthine/xanthine oxidase system and monitored by the production of nitroblue tetrazolium (NBT), using a slight modification of a published procedure (Valentao et al., 2001[48]). The reaction mixtures containing five different concentrations of the extracts, 0.5 mM xanthine, and 0.5 mM NBT were incubated at room temperature for 2 min. The reaction was initiated by the addition of xanthine oxidase (50 mU/ml). After standing for 20 min, the absorbance was recorded at 560 nm using an ELISA reader. The concentration of extract providing 50 % inhibition (IC_50_) was calculated from a plot of the inhibition percentage against the extract concentration. The experiments were conducted in triplicate. BHT, Trolox and allopurinol were used as the positive controls.

### Xanthine oxidase inhibition assay

Xanthine oxidase inhibition properties were evaluated by measuring the formation of uric acid from xanthine at room temperature. The reaction mixtures consisted of five different concentrations of the plant extracts in 100 µl of 200 mM potassium phosphate buffer (pH 7.5) containing 1 mM EDTA, 0.5 mM xanthine, and 50 mU/ml xanthine oxidase. BHT, Trolox and allopurinol were used as positive controls. The change in absorbance at 290 nm was recorded over time using a UV spectrophotometer. Activity was expressed as IC_50_, and was defined as the concentration required for scavenging 50 % of the uric acid in the solution. All experiments were performed in triplicate.

### Hydrogen peroxide scavenging assay

Hydrogen peroxide scavenging activity was determined according to the method described by Muller (1995[32]). 100 µl of 0.1 M phosphate buffer (pH 5.0) and the plant extracts were combined together in a 96-well plate. 20 µl of H_2_O_2_ was added to the mixture, and incubated at 37 °C for 5 min. After incubation, 30 µl of 1.25 mM ABTS and 30 µl of peroxidase (1 unit/ml) were added to the mixture and then incubated for 10 min at 37 °C. The absorbance was read using an ELISA reader at 405 nm. Scavenging activity was expressed as IC_50_, and defined as the concentration required for scavenging 50 % of the hydrogen peroxide in the solution. The experiments were performed in triplicate. BHT, Trolox and allopurinol were used as the positive controls.

### Determination of total phenolic content

The total polyphenolic compounds present in the plant extracts were quantified using a protocol adapted from Chandler and Dodds (1983[7]). 1 ml of plant extract was added into a test tube containing a mixture of 1 ml of 95 % ethanol, 5 ml of distilled water, and 0.5 ml of 50 % Folin-Ciocalteu reagent. The mixture was then allowed to react for 5 min, after which 1 ml of 5 % Na_2_CO_3_ was added. The mixture was incubated in a dark room for 1 hour at room temperature, and the absorbance was recorded at a wavelength of 725 nm using an ELISA plate reader. Tannin acid was used as a standard, and the total phenolic content of the plant extract tested was expressed as a tannin acid equivalent (TAE, mg tannin acid/g extract). The data was reported as mean ± SD for at least three independent replications.

### Determination of total flavonoid content

Total flavonoid content was determined using a colorimetric method described by Jia et al. (1999[24]) with minor modifications. 1 ml of plant extract was added to a volumetric flask containing 1 ml of 5 % (w/v) sodium nitrite and incubated for 6 min at room temperature. This was followed by the incorporation of 1 ml of 10 % (w/v) aluminum nitrate into the reaction mixture so as to allow for the formation of a flavonoid–aluminum complex. After 6 min, 10 ml of 4.3 % (w/v) NaOH was added and the total solution was made up to 25 ml with distilled water. After a 15 minute incubation at room temperature, the final solution was mixed thoroughly again and the absorbance measured against a blank at 510 nm using an ELISA plate reader. The total flavonoid content of plant extracts was expressed as a rutin equivalent (RE, mg rutin/g extract). The data was reported as means ± SD for at least three independent replications.

### Assays conducted using electron spin resonance (ESR) spectroscopy

#### DPPH radical scavenging assay

DPPH radical scavenging activity was measured using the method described by Nanjo et al. (1996[33]). An ethanolic solution of 60 µl of each sample (or ethanol itself as the control) was added to 60 µl of DPPH (60 µmol/l) in ethanol. After mixing vigorously for 10 sec, the solution was transferred into a 100 µl Teflon capillary tube and fitted into the cavity of the ESR spectrometer (JES-FA machine, JOEL, Tokyo, Japan). The spin adduct was measured on an ESR spectrometer exactly 2 min later. The measurement conditions used were: central field 3475 G, modulation frequency 100 kHz, modulation amplitude 2 G, microwave power 5 mW, gain 6.3×10^5^ and a temperature of 298 K.

#### Hydroxyl radical scavenging assay

Hydroxyl radicals were generated by the Fenton reaction, and reacted rapidly with nitrone spin trap DMPO; the resultant DMPO-OH adducts was detectable with an ESR spectrometer (Rosen and Rauckman, 1984[38]). The ESR spectrum was recorded 2.5 min after addition of a phosphate buffer solution (pH 7.4) with 0.3 M DMPO 0.2 ml, 10 mM FeSO_4_ 0.2 ml and 10 mM H_2_O_2_ 0.2 ml using an ESR spectrometer set at the following conditions: central field 3475 G, modulation frequency 100 kHz, modulation amplitude 2 G, microwave power 1 mW, gain 6.3×10^5^ and a temperature of 298 K.

#### Alkyl radical scavenging assay

Alkyl radicals were generated by AAPH. The PBS (pH 7.4) reaction mixtures containing 10 mmol/l AAPH, 10 mmol/l 4-POBN and indicated concentrations of tested samples, were incubated at 37 °C in a water bath for 30 min (Hiramoto et al., 1993[19]) and then transferred to a 100 µl Teflon capillary tube. The spin adduct was recorded on JES-FA ESR spectrometer. Measurement conditions: central field 3475 G, modulation frequency 100 kHz, modulation amplitude 2 G, microwave power 10 mW, gain 6.3×10^5^ and a temperature of 298 K.

### Cell culture

To study the inhibition effect of EtOAc fraction of CN-13 extract on H_2_O_2_-mediated DNA damage, we used the L5178 mouse T-cell lymphoma cell line (L5178Y-R). The mouse lymphoma cell line (L5178Y-R) was maintained at 37 °C in an incubator with a humidified atmosphere of 5 % CO_2_. Cultures were grown in RPMI 1640 medium supplemented with 10 % (v/v) heat inactivated fetal bovine serum (FBS), penicillin (100 U/ml) and streptomycin (100 µg/ml).

### Determination of DNA damage (Comet assay)

The alkaline comet assay was conducted according to the method described by Singh et al. (1995[44]) with a slight modification. The number of cultured cells was adjusted to 4 × 10^4^ cells/ml. The cells were incubated with each sample at concentrations ranging from 6.25 to 50 µg/ml for 30 min at 37 °C. After pre-incubation, the cells were centrifuged at a 3000 rpm for 5 min and then washed using phosphate buffered saline (PBS). Following this, the cells were resuspended in PBS with 50 µM H_2_O_2_ for 5 min on ice. The untreated control cells were resuspended only in PBS without H_2_O_2_. The cells were washed with 1 ml PBS and centrifuged. The cell suspension was mixed with 100 µl of 0.7 % low melting point agarose (LMPA), and added to 1.0 % normal melting point agarose (NMPA)-coated slides. After incubation at 4 °C for 10 min, the slides were covered with another 100 µl of 0.7 % LMPA and incubated at 4 °C for 40 min to allow for solidification of the agarose. Later the slides were immersed in lysis solution (2.5 M NaCl, 100 µM EDTA, 10 mM Tris, 1 % sodium laurylsarcosine and 1 % Triton X-100) at 4 °C for an hour. The slides were then unwinded and electrophoresis was applied with an electric current of 25 V/300 mA for 20 min. The slides were neutralized in 0.4 M Tris buffer (pH 7.5) for 10 min twice and dehydrated with 70 % ethanol. The percentage of fluorescence in the DNA tail of each cell (tail intensity, TI; 50 cells from each of two replicate slides) on the ethidium bromide stained slides was measured by image analysis (Kinetic Imaging, Komet 5.0, UK) and fluorescence microscopy (LEICA DMLB, Germany).

### Statistical analysis

All data was analyzed using the SPSS package for Windows (Version 10). Values were expressed as mean ± standard error (SE). The mean values of the tail intensity from each treatment were compared using one-way analysis of variance (ANOVA) followed by Duncan’s multiple range tests. P-value of less than 0.05 was considered significant.

## Result and Discussion

Free radicals are harmful by-products generated during normal cellular metabolism, which could initiate oxidative damage in the body (Abidi and Ali, 1999[1]; Halliwell and Aruoma, 1991[14]). Antioxidants are believed to play a significant role in the body’s defense system against free radical damage. Recently, numerous studies have described antioxidant compounds with radical-scavenging activity present in fruits, vegetables, herbs and cereals extracts (Gray et al., 2002[12]; Nuutila et al., 2003[34]; Hou et al., 2005[22]). The DPPH radical scavenging activity, superoxide radical scavenging activity, H_2_O_2_ scavenging activity and xanthine oxidase inhibition ability of 80 % ethanol extracts of 16 plants are shown in Table 2(Tab. 2). DPPH is the agent of choice for many similar studies in evaluating the free radical scavenging activity of natural compounds (Shimada et al., 1992[41]). As shown in Table 2(Tab. 2), 6 plant extracts (CN-7, -9, -10, -12, -13, and -14 extracts) exhibited IC_50_ values below 60 µg/ml, indicating good potential as DPPH radical scavengers. Of all the plant extracts tested, CN-13 showed greater capacity (IC_50_ value of 20.9 µg/ml) to scavenge the DPPH radical when compared to that of BHA (IC_50_ value of 22.7 µg/ml).

Superoxide radicals were generated by the hypoxanthine-xanthine oxidase and the NBT system. The decrease in absorbance at 550 nm with the presence of an antioxidant indicated the consumption of superoxide radicals in the reaction mixture. Of the tested samples, CN-7, -8, -12, -13, -14, and -15 extracts showed higher scavenging properties (IC_50_ value of 14.4, 20.3, 15.5 13.3, 13.5, and 28.2 µg/ml, respectively) against the superoxide radical than the other extracts. These values were higher than that of Trolox and Allpurinol, with an IC_50_ value of 189.9 and 22.7 µg/ml.

Hydrogen peroxide can be formed *in vivo* by an antioxidant enzyme such as superoxide dismutase. It is capable of crossing membranes and may slowly oxidize a number of compounds. CN-13 and -12 plant extracts showed similar scavenging properties (IC_50_ value of 41.7 and 48.4 µg/ml) against H_2_O_2_. Moreover, CN-9, -10, -14, and -15 extracts showed relatively higher H_2_O_2_ scavenging activity, with a reported IC_50_ value of 76.1, 71.4, 56.6, and 86.8 µg/ml, respectively.

Xanthine oxidase also acts as an important biological source of oxygen-derived free radicals that contribute to oxidative damage in living tissues, resulting in many pathological processes such as inflammation, atherosclerosis, cancer and aging (Chiang et al., 1994[8]; Sweeney et al., 2001[45]). CN-7, -12, 14, and -15 plant extracts showed xanthine oxidase inhibition activity, with an IC_50_ value of 448.1, 443.9, 653.5, and 296.9 µg/ml, respectively. Previous studies have reported high antioxidant and radical-scavenging activities in plants (Amarowicz et al., 2004[2]; Miliauskas et al., 2004[31]; Silva et al., 2005[43]; Kumaran and Karunakaran, 2007[30]). Of particular note is that the plant extracts of *Quercus* and *Machilus* species have been shown to exhibit profound DPPH radical scavenging activities (Hou et al., 2003[21]; Rakic et al., 2007[37]).

Polyphenolic compounds are distributed widely throughout plants and seaweeds, and have demonstrated profound antioxidative properties. These properties have been found to represent a variety of ROS scavenging and lipid peroxidation inhibition activities (Athukorala et al., 2003[4]; Scalbert et al., 2005[39]; Kang et al., 2005[27]; Tadhani et al., 2007[46]). The phenolic contents of 80 % EtOH-plant extracts determined, are shown in Table 3(Tab. 3). CN-13, -15 and -9 extracts showed relatively higher phenolic contents of 482.0, 519.1 and 462.3 mg TAE/g of plant extracts, respectively (Table 3(Tab. 3)), when compared to other 80 % EtOH-plant extracts. The antioxidant and radical scavenging properties of CN-13, -15 and -9 plant extracts were also reported to be higher than that of the other plant extracts tested (Table 2(Tab. 2)). It was of importance to examine the correlation between the content of total polyphenols present in the plant extract sample and its antioxidant potential since some authors have reported that no correlation exists between the presence of these antioxidant compounds and the radical scavenging capacity of the tested sample (Yu et al., 2002[51]). The results obtained in this study do not support these claims. These data are in accordance with other published reports showing that a high total phenol content in the test sample increased its antioxidant properties (Holasova et al., 2002[20]; Tepe and Sokmen, 2007[47]; Kumaran and Karunakaran, 2007[30]).

We examined the flavonoid contents of the 80 % EtOH extracts from 16 plant species as shown in Table 3(Tab. 3). The flavonoid contents of CN-13, -15 and -9 extracts were higher (389.6, 358.4 and 365.1 mg RE/g plant extract, respectively) than that of the other extracts tested. Flavonoids, one of the most diverse and widespread group of natural compounds, are probably the most natural phenolics (Shimoi et al., 1996[42]). Flavonoids have been reported to be efficient antioxidants by scavenging oxygen radicals (Hanasaki et al., 1994[15]) and possessing anti-cancer, hypolipidaemic, anti-ageing, and anti-inflammatory activities (Cody et al., 1988[9]). CN-13, -15 and -9 plant extracts were shown to be strong radical scavengers, indicating that active compounds of varying polarity could be present in these plants. The high antioxidant activities of these plants might be attributable to their flavonoid and phenolic contents.

**Figure 1:**
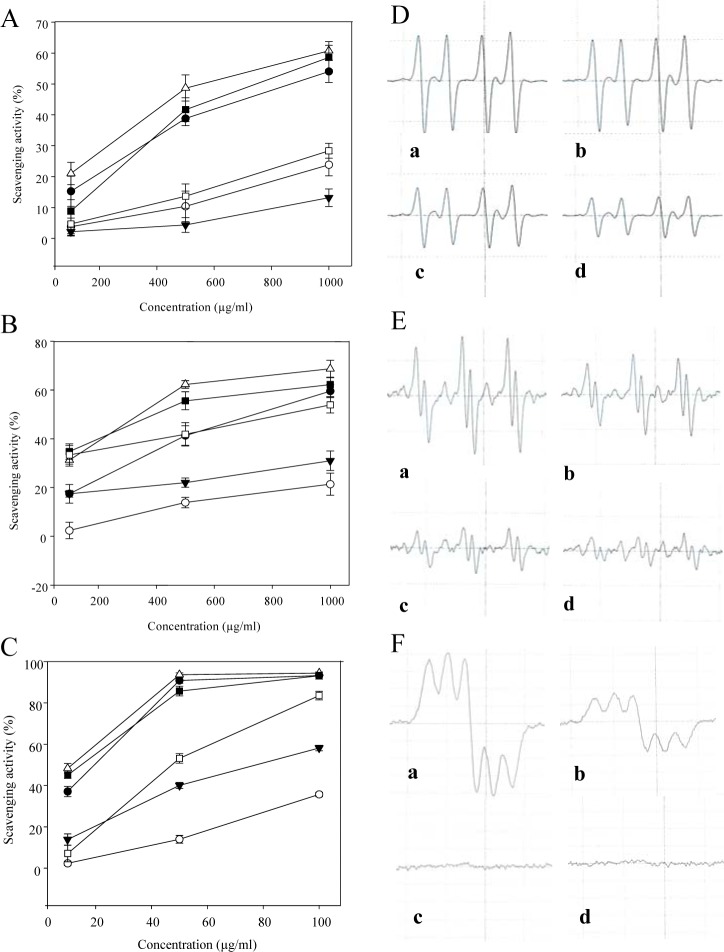
(A) Hydroxyl, (B) Alkyl, and (C) DPPH radical scaveng-ing activity of the extract and its various fractions of CN-13 ethanol extract ( ● , EtOH extract; ○ *n*-hexane fraction; ▼ CH_2_Cl_2_ fraction; ∆ , EtOAc fraction; ■ BuOH fraction; □ water fraction). Mean ± SE of determinations was deducted from triplicate experiments. (D) ESR spectrum obtained in the Fenton reaction system at various concentrations of the EtOAc fraction of CN-13 ethanol extract a, control; b, 100 µg/ ml; c, 500 µg/ml; d, 1000 µg/ml. (E) ESR spectrum observed during the incubation of AAPH with 4-POBN at various concentrations of the EtOAc fraction of CN-13 ethanol extract (a, control; b, 100 µg/ ml; c, 500 µg/ml; d, 1000 µg/ml). (F) ESR spectrum obtained in an ethanol solution of 60 µmol/l DPPH at various concentrations of EtOAc fraction from CN-13 ethanol extract (a, control; b, 10 µg/ml; c, 50 µg/ml; d, 100 µg/ ml).

Of the tested samples, CN-13 extract evidenced the highest antioxidant properties, as well as increased polyphenol and flavonoid contents. Therefore, the CN-13 extract was selected for use in further experiments, and resolved into different solvent fractions. Hydroxyl radicals, generated in the Fe^2+^/H_2_O_2_ system, were trapped by DMPO, forming a spin adduct detected by the ESR spectrometer. A typical 1:2:2:1 ESR signal of the DMPO-OH adduct was observed as shown in Figure 1A(Fig. 1) and D(Fig. 1). In addition, background signals were also present (Figure 1D(Fig. 1)), which could be attributed to the paramagnetic impurities contained in unpurified commercial DMPO (Rosen and Rauckman, 1984[38]). The height of the third peak of the spectrum represented the relative amount of DMPO-OH adduct as was shown on the ESR spectrum. As shown in Figure 1A(Fig. 1), it was observed that the hydroxyl radical scavenging activities of 80 % EtOH extract (CN-13) and its solvent fractions (*n*-hexane, CH_2_Cl_2_, EtOAc, BuOH and water fraction) were 54.1 %, 23.9 %, 13.2 %, 60.8 %, 58.6 % and 28.4 % at 1000 µg/ml, respectively. Almost all the extracts scavenged the hydroxyl radicals, and the scavenging activities increased with increasing concentrations of the extract and fractions. 

The alkyl radical spin adduct of 4-POBN/free radicals generated from AAPH at 37 °C for 30 min and the decrease of ESR signals were observed with a dose increment of EtOAc fraction from CN-13 extract (Figure 1B(Fig. 1) and E(Fig. 1)). A comparison of the different solvent fractions of CN-13 showed that the EtOAc and BuOH fractions scavenged more than 60 % of the free radicals generated, with values of 68.7 % and 62.2 % at 1000 µg/ml, respectively (Figure 1B(Fig. 1)). Moreover, the EtOAc fractions exhibited strongest scavenging activity at the lowest concentration (500 µg/ml).

The ESR spectrum of the DPPH radical scavenging activity of the EtOAc fraction of CN-13 extract is shown in Figure 1C(Fig. 1) and E(Fig. 1). The radical scavenging activity demonstrated was concentration-dependent. DPPH radical scavenging activity of an 80 % EtOH extract, *n*-hexane, CH_2_Cl_2_, EtOAc, BuOH and water fractions were calculated to be 90.9 %, 14.0 %, and 40.1 %. 93.7 %, 85.8 % and 53.2 % at 50 µg/ml, respectively (Figure 1C(Fig. 1)). Generally, the DPPH signals decrease when the odd electron of the DPPH radical is paired. The results indicated that 80 % EtOH extract, EtOAc and BuOH fractions were found to possess DPPH radical scavenging activity by pairing the odd electron of DPPH radicals.

The total phenolics in 80 % EtOH extracts and the fractions of CN-13 were determined according to the Folin-Ciocalteu method and expressed as tannin acid equivalent (TAE). As shown in Table 4(Tab. 4), the greatest content of total phenolic constituents was present in the EtOAc fraction (883.5 mg TAE/g extract), followed by BuOH fraction (557.2 mg TAE/g extract), 80 % EtOH extract (428.0 mg TAE/g extract), CH2Cl2 fraction (168.6 mg TAE/g extract), water fraction (130.8 mg TAE/g extract) and *n*-hexane fraction (112.0 mg TAE/g extract). Furthermore, the EtOAc fraction showed the presence of the highest flavonoid contents (820.1 mg RE/g extract) compared to the other fractions (Table 4(Tab. 4)). A correlation between the total phenolic, flavonoid and antioxidant potential was observed in the present study.

DNA damage is one of the most sensitive biological markers for the evaluation of oxidative stress, and illustrates the imbalance between free radical generation and the efficiency of the antioxidant system (Gutteridge, 1995[13]; Kassie et al., 2000[28]). Hydrogen peroxide (H_2_O_2_) is a reactive oxygen species and is used to induce DNA damage in cells. Therefore, we investigated the effect of varying concentrations (6.25, 12.5, 25 and 50 µg/ ml) of H_2_O_2_ on DNA damage in the presence of an EtOAc fraction of CN-13 extract using the comet assay. The protective effect of the EtOAc fraction on H_2_O_2_-induced DNA damage in L5178Y-R cell line is shown in Table 5(Tab. 5).

**Figure 2:**
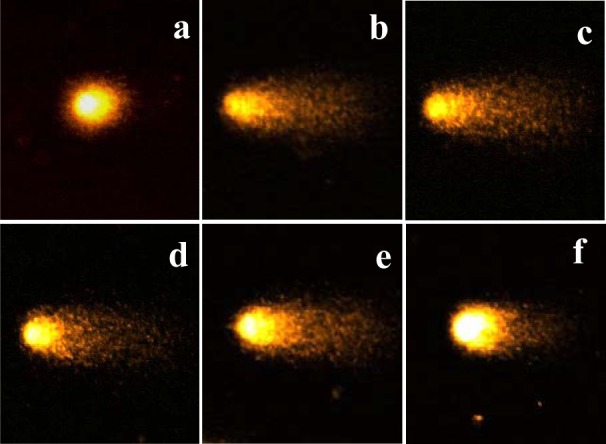
Photomicrographs of DNA damage and migration observed under the various concentration of EtOAc fraction of CN-13 ethanol extract. a, Negative control; b, L5178Y-R cell lines treated with 50 µM H_2_O_2_; c, L5178Y-R cell lines treated with 6.25 µg/ml EtOAc fraction + 50 µM H_2_O_2_; d, L5178Y-R cell lines treated with 12.5 µg/ml EtOAc fraction + 50 µM H2O2; e, L5178Y-R cell lines treated with 25 µg/ml EtOAc fraction + 50 µM H_2_O_2_; f, L5178Y-R cell lines treated with 50 µg/ml EtOAc fraction + 50 µM H_2_O_2_

The addition of H_2_O_2_ to the cell culture medium in the absence of the plant extract resulted in a 43.2 % DNA damage. However, the addition of the EtOAc fraction resulted in a dose-dependent decrease in DNA damage. Of particular note is that the EtOAc fraction showed good inhibitory (58.6 %) effects against DNA damage at 50 µg/ml. Furthermore, we identified photomicrographs of different DNA migration profiles, when treated with concentrations of samples and only H_2_O_2_. In the group treated with only H_2_O_2_ (Figure 2b(Fig. 2)), the DNA was completely damaged and significant increases in the amounts of tail DNA were observed when compared to that of untreated cells (Figure 2a(Fig. 2)). However, when cells were treated with the sample, the amounts of tail DNA were increasingly decreased with increasing concentrations of the EtOAc fraction (Figure 2(Fig. 2)). The results of several previous studies have indicated that increases in the content of a variety of materials (environmental pollutants, radiation, dietary habits and various chemicals) could induce DNA damage, which can result in conditions such as cancer and heart disease (Hertog et al., 1993[18]; Hartmann et al., 1995[17]; Singh et al., 1995[44]). Moreover, a host of researchers have investigated the inhibition of DNA damage affected by food materials such as tea (Zhang et al., 2002[54]), juice (Park et al., 2003[35]), plant extract (Yen et al., 2001[50]; Zhu and Loft, 2001[55]), flavonoids (Senthilmohan et al., 2003[40]), and aquatic animals (Janssens et al., 2002[23]). Cells in the human body are basically under continuous attack by physical agents (such as solar radiation), a variety of chemical compounds, and ROS, all of which can induce DNA damage. If the DNA damage remains unresolved, a cascade of biological consequences can be initiated within the cell (Bagchi et al., 2000[5]).

## Conclusion

On the basis of the results of this study, it was concluded that 80 % EtOH extracts of CN-13 showed strong antioxidant properties against the inhibition of the DPPH radical, superoxide radical and hydrogen peroxide (H_2_O_2_). The EtOAc fraction of 80 % EtOH extract from CN-13 exhibited greater DPPH radical scavenging activity than the EtOH extract as well as the other fractions. In addition, the EtOAc fraction showed a greater inhibitory effect on the DNA damage induced by H_2_O_2_. Therefore, the EtOAc fraction of an 80 % EtOH extract from CN-13 proves usefulness in both the food and pharmaceutical industries. Further studies are required in order to isolate and identify the antioxidant components of the EtOAc fraction.

## Notes

Giannae Ahn (Department of Marine Bio-Food Sciences, Chonnam National University, Yeosu 550-749, Republic of Korea; Tel.: +82-61-659-7213; e-mail: gnahn@jnu.ac.kr) and Kil-Nam Kim (Jeju Center, Korea Basic Science Institute (KBSI), Jeju 690-140, Republic of Korea; Tel.: +82-64-800-4933; e-mail: knkim@kbsi.re.kr) contributed equally as corresponding authors.

## Acknowledgement

This research was supported by the project fund (C35290) to D. Kim from Korea Basic Science Institute.

## Figures and Tables

**Table 1:**
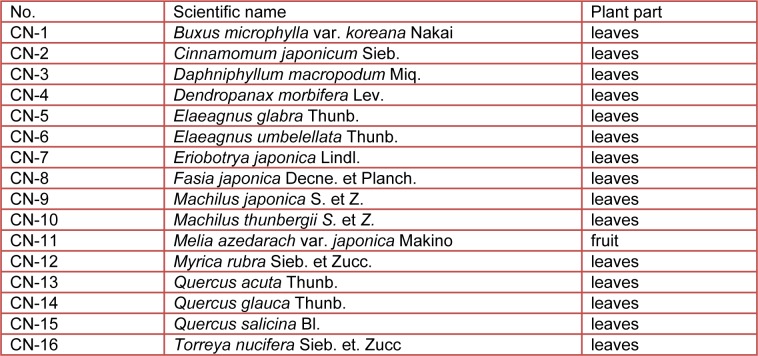
Plant species used in this study

**Table 2:**
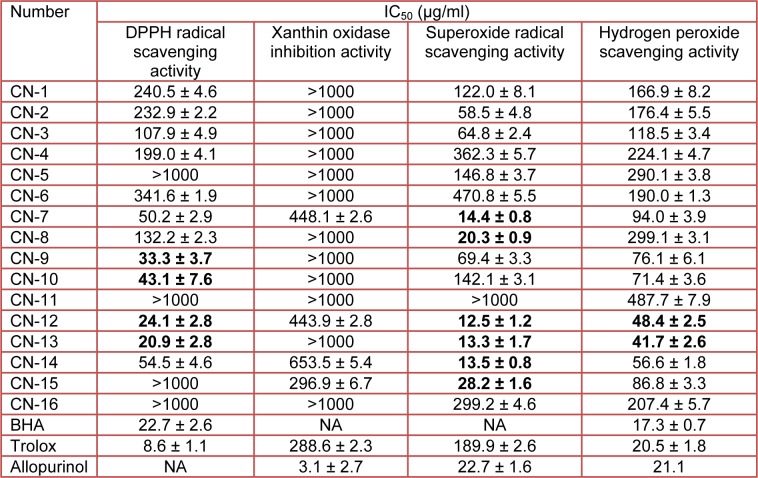
A comparison of the antioxidant properties of the ethanol extracts of 16 species plants from Jeju Island

**Table 3:**
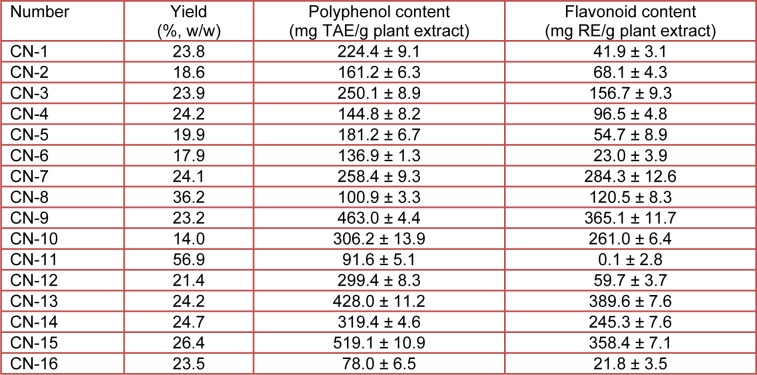
The yield, total polyphenols and flavonoids contents of ethanol extracts of 16 species from Jeju Island

**Table 4:**
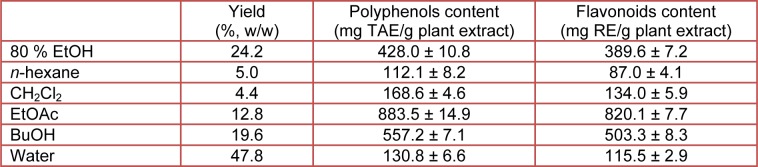
The yield, total polyphenols and flavonoids contents of CN-13 ethanol extract and its various fractions

**Table 5:**
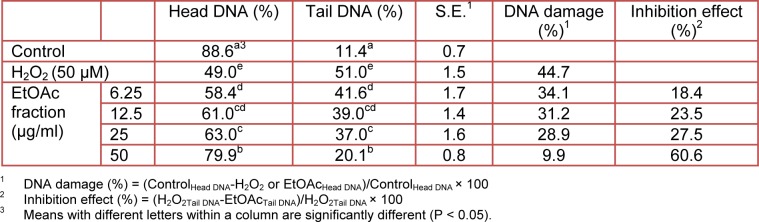
The effect of* in vitro* supplementation of varying concentrations of an EtOAc fraction of CN-13 ethanol extract on H_2_O_2_-induced mouse lymphoma (L5178Y-R) cell damage

## References

[1] Abidi S, Ali A (1999). Role of ROS modified human DNA in the pathogenesis and etiology of cancer. Cancer Lett.

[2] Amarowicz R, Pegg RB, Rahimi-Moghaddam P, Barl B, Weil JA (2004). Free-radical scavenging capacity and antioxidant activity of selectedplant species from the Canadian prairies. Food Chem.

[3] Ames B (1998). Micronutrients prevent cancer and delay aging. Toxicol Lett.

[4] Athukorala Y, Lee KW, Song CB, Shin TS, Cha YJ, Shahidi F (2003). Potential antioxidant activity of marine red alga Grateloupia filicina extract. J Food Lipids.

[5] Bagchi D, Bagchi M, Stohs SJ, Das DK, Ray SD, Kuszynski CA (2000). Free radicals and grape seed proanthocyanidin extract: importance in human health and disease prevention. Toxicology.

[6] Blois MS (1958). Antioxidant determinations by the use of a stable free radical. Nature.

[7] Chandler SF, Dodds JH (1983). The effect of phosphate, nitrogen and sucrose on the production of phenolics and solasidine in callus cultures of Solanum laciniatum. Plant Cell Rep.

[8] Chiang HC, Lo YJ, Lu FJ (1994). Xanthine oxidase inhibitions from leaves of Alsophila spinulosa (Hook) Tryon. J Enzym Inhib.

[9] Cody V, Middleton E, Harborne JB, Bertz A (1988). Plant flavonoids in biology and medicine II: biochemical, cellular and medicinal properties.

[10] Cox DA, Cohen ML (1996). Effects of oxidized low density lipoproteins on vascular contraction and relaxation. Pharmacol Rev.

[11] Finkel T, Holbrook NJ (2000). Oxidants, oxidative stress and the biology of ageing. Nature.

[12] Gray DA, Clarke MJ, Baux C, Bunting JP, Salter AM (2002). Antioxidant activity of oat extracts added to human LDL particles and in free radical trapping assays. J Cereal Sci.

[13] Gutteridge JMC (1995). Lipid peroxidation and antioxidants as biomarker of tissue damage. Clin Chem.

[14] Halliwell B, Aruoma OI (1991). DNA damage by oxygen-derived species. FEBS Lett.

[15] Hanasaki Y, Ogawar S, Fukui S (1994). The correlation between active oxygen scavenging and antioxidative effects of flavonoids. Free Rad Biol Med.

[16] Harman D (1994). Free radical theory of aging, increasing the functional life span. Ann NY Acad Sci.

[17] Hartmann A, Herkommer K, Gluck M, Speit G (1995). DNA-damaging effect of cyclophosphamide on human blood cells in vivo and in vitro studied with the single-cell gel test (Comet assay). Environ Mol Mutagen.

[18] Hertog MGL, Feskens EJM, Hollman PCH, Katan MB, Kromhout D (1993). Dietary antioxidants flavonoids and the risk of coronary heart disease: the zutphen elderly study. Lancet.

[19] Hiramoto K, Johkoh H, Sako KI, Kikugawa K (1993). DNA breaking activity of the carbon-centered radical generated from 2,2´-azobis(2-amidinopropane) hydrochloride (AAPH). Free Rad Res Commun.

[20] Holasova M, Fiedlerova V, Smrcinova H, Orsak M, Lachman J, Vavreinova S (2002). Buckwheat - the source of antioxidant activity in functional foods. Food Res Int.

[21] Hou WC, Lin RD, Cheng KT, Hung YT, Cho CH, Chen CH (2003). Free radical-scavenging activity of Taiwanese native plants. Phytomedicine.

[22] Hou WC, Lin RD, Lee TH, Huang YH, Hsu FL, Lee MH (2005). The phenolic constituents and free radical scavenging activities of Gynura formosana Kiamnra. J Sci Food Agric.

[23] Janssens BJ, Le Gall R, Rees JF (2002). Peroxide-triggered erythrocytes haemolysis as a model for the study of oxidative damage in marine fishes. J Fish Biol.

[24] Jia Z, Mengcheng T, Wu J (1999). The determination of flavonoid contents in mulberry and their scavenging effects on superoxide radicals. Food Chem.

[25] Kahl R (1994). Synthetic antioxidants: biochemical actions and interference with radiation, toxic compounds, chemical mutagens and chemical carcinogens. Toxicology.

[26] Kahl R, Kappus H (1993). Toxicology of the synthetic antioxidants BHA and BHT in comparison with the natural antioxidant vitamin E. Z Lebensm Unters Forsch.

[27] Kang KA, Lee KH, Chae S, Zhang R, Jung MS, Lee Y (2005). Eckol isolated form Ecklonia cava attenuates oxidative stress induced cell damage in lung fiblroblast cells. FEBS Lett.

[28] Kassie F, Parzefall W, Knasmuller S (2000). Single cell gel electrophoresis assay: a new technique for human biomonitoring studies. Mutat Res.

[29] Kinsella JE, Frackel E, German B, Kanner J (1993). Possible mechanisms for the protective role of antioxidants in wine and plant foods. Food Technol.

[30] Kumaran A, Karunakaran RJ (2007). In vitro antioxidant activities of methanol extracts of five Phyllanthus species from India. LWT-Food Sci Technol.

[31] Miliauskas G, Venskutonis PR, Beek TA (2004). Screening of radical scavenging activity of some medicinal and aromatic plant extracts. Food Chem.

[32] Muller HE (1995). Detection of hydrogen peroxide produced by microorganism on ABTS-peroxidase medium. Zbl Bakteriol Mikrobiol Hyg.

[33] Nanjo F, Goto K, Sto R, Suzuki M, Sakai M, Hara Y (1996). Scavenging effects of tea catechens and their derivatives on 1,1,-diphenyl-2-picrylydrazyl radical. Free Rad Biol Med.

[34] Nuutila AM, Puuponen-Pimiä T, Aarni M, Oksman-Caldentey KM (2003). Comparison o antioxidant activities of onion and garlic extracts by inhibition of lipid peroxidation and radical scavenging activity. Food Chem.

[35] Park YK, Park E, Kim JS, Kang MH (2003). Daily grape juice consumption reduces oxidative DNA damage and plasma free radical levels in healthy Koreans. Mutat Res.

[36] Pietta PG (2000). Flavonoids as antioxidant. J Nat Prod.

[37] Rakić S, Petrović S, Kukić J, Jadranin M, Tešević V, Povrenović D (2007). Influence of thermal treatment on phenolic compounds and antioxidant properties of oak acorns from Serbia. Food Chem.

[38] Rosen GM, Rauckman EJ (1984). Spin trapping of superoxide and hydroxyl radical. Meth Enzymol.

[39] Scalbert A, Manach C, Morand C, Remesy C (2005). Dietary polyphenols and the prevention of diseases. Crit Rev Food Sci Nutr.

[40] Senthilmohan ST, Zhang J, Stanley RA (2003). Effects of flavonoid extract Enzogenol with vitamin C on protein oxidation and DNA damage in older human subjects. Nutr Res.

[41] Shimada K, Fujikawa K, Yahara K, Nakamura T (1992). Antioxidative properties of xanthan on the autoxidation of soybean oil in cyclodextrin. J Agric Food Chem.

[42] Shimoi K, Masuda S, Shen B, Furugori B, Kinae N (1996). Radioprotective effect of antioxidative plant flavonoids in mice. Mutat Res.

[43] Silva CG, Herdeiro RS, Mathias CJ, Panek AD, Silveira CS, Rodrigues VP (2005). Evaluation of antioxidant activity of Brazilian plant. Pharm Res.

[44] Singh NP, Graham MM, Singh V, Khan A (1995). Induction of DNA single-strand breaks in human lymphocytes by low doses of X-rays. Int J Radiat Biol.

[45] Sweeney AP, Wyllie SG, Shalliker RA, Markhan JL (2001). Xanthine oxidase inhibitory activity of selected Australian native plants. J Ethnopharmacol.

[46] Tadhani MB, Patel VH, Subhash R (2007). In vitro antioxidant activities of Stevia rebaudiana leaves and callus. J Food Comp Anal.

[47] Tepe B, Sokmen A (2007). Screening of the antioxidative properties and total phenolic contents of three endemic Tanacetum suspecies from Turkish flora. Biores Technol.

[48] Valentao P, Fernandes E, Carvalho F, Andrade PB, Seabra RM, Bastos ML (2001). Antioxidant activity of Centaurium erythraea infusion evidenced by its superoxide radical scavenging and xanthine oxidase inhibitory activity. J Agric Food Chem.

[49] Wanasundara PK, Shahidi F (1996). Optimization of hexametaphosphate-assisted extraction of flaxseed proteins using response surface methodology. J Food Sci.

[50] Yen GC, Chen HY, Peng HH (2001). Evaluation of the cytotoxicity, mutagenicity and antimutagenicity of emerging edible plants. Food Chem Toxicol.

[51] Yu L, Haley S, Perret J, Harris M, Wilson J, Qian M (2002). Free radical scavenging properties of wheat extracts. J Agric Food Chem.

[52] Yuting C, Rongliang Z, Zhongjian J, Yong J (1990). Flavonoids as superoxide scavengers and antioxidants. Free Rad Biol Med.

[53] Zhang D, Yasuda T, Yu Y, Zheng P, Kawabata T, Ma Y (1996). Ginseng extract scavenges hydroxyl radical and protects unsaturated fatty acids from decomposition caused by iron mediated lipid peroxidation. Free Rad Biol Med.

[54] Zhang H, Spitz MR, Tomlinson GE, Schabath MB, Minna JD, Wu X (2002). Modification of lung cancer susceptibility by green tea extract as measured by the Comet assay. Cancer Detect Prev.

[55] Zhu CY, Loft S (2001). Effects of Brussels sprout extracts on hydrogen peroxide-induced DNA strand breaks in human lymphocytes. Food Chem Toxicol.

